# Nasal-Ocular Reflexes and Their Role in the Management of Allergic Rhinoconjunctivitis With Intranasal Steroids

**DOI:** 10.1097/1939-4551-4-S1-S1

**Published:** 2011-01-15

**Authors:** Fuad M Baroody, Robert M Naclerio

**Affiliations:** 1 Department of Surgery, Section of Otolaryngology-Head and Neck Surgery, The University of Chicago Medical Center, Chicago, IL

**Keywords:** Nasal-ocular reflex, allergic rhinoconjuctivitis, nasal challenge, intranasal steroids

## Abstract

Allergic rhinitis is a common disorder and involves the reaction to environmental allergens with resultant nasal and eye symptoms. The pathophysiologic mechanisms of the eye symptoms in allergic conjunctivitis include a direct effect on the eye by deposited allergen and indirect effects related to the deposition of allergen in the nasal mucosa. One of these proposed mechanisms is the existence of a nasal-ocular reflex whereby the nasal allergic reaction leads to an afferent reflex response, the efferent limb of which results in eye symptoms. Among the treatments available for allergic rhinitis, intranasal steroids are most efficacious for nasal symptoms and have also shown sizeable efficacy related to eye symptoms. We speculated that the effect of intranasal steroids on eye symptoms in allergic rhinitis was related to their inhibition of the nasal-ocular reflex and present data previously generated from our laboratory to support this assumption in a nasal challenge model.

## 

Allergic rhinitis is characterized by an IgE-mediated reaction to environmental allergens. It is a common disorder affecting up to 40 million Americans with adult prevalence estimates ranging from 10 to 30% and pediatric prevalence estimates as high as 40% rendering it the most common chronic condition in children.1 Several reports also support substantial increases in the prevalence of allergic rhinoconjunctivitis (AR) in developed countries in recent decades making it an important health problem[1-3].

Although often referred to as allergic rhinitis, this disease actually involves eye symptoms in addition to the nasal symptoms, hence the more appropriate term, AR. In fact, recent epidemiologic data has shown that ocular symptoms, defined as 'episodes of watery, itchy eyes,' affected 40% of the adult population of the United States during a 12-month period[4]. Another study showed that the incidence of conjunctivitis was high (~88%) in patients experiencing allergic rhinitis during the cypress pollen season[5]. Ocular symptoms are not only common, but also distressing for allergy sufferers, with more than 50% stating that watery and red/itchy eyes were moderately to extremely bothersome in the Allergies in America survey[6]. Furthermore, in 15% of sufferers, the ocular component of the allergic hypersensitivity reaction was the most bothersome symptom[6].

In addition to the typical nasal and eye symptoms, AR leads to a significant impairment of the quality of life of its sufferers when measured by both generic and specific quality of life instruments[7,8]. Also associated with the disease are fatigue and daytime sleepiness,[7,9] reduced work productivity,[10-12] impaired cognitive functioning,[13,14] reduced learning abilities,[15] and impaired sleep[16].

The high prevalence of this benign, but chronic, condition, its adverse effects on quality of life, work performance, and productivity on the job, and the treatments sought by the sufferers to alleviate the symptoms all result in significant health care expenditure. The healthcare costs related to AR have been reported to be US$5.9 billion annually in the United States, with medication use accounting for 25% of these costs[17]. 

## Pathophysiology Of AR

The pathophysiologic mechanisms involved in AR start by the sensitization of the nasal mucosa to a certain allergen that involves multiple interactions among antigen presenting cells, T lymphocytes, and B cells and lead to the production of antigen-specific IgE antibodies, which then localize to mast cells and basophils. Subsequent exposure leads to crosslinking of specific IgE receptors on mast cells and their resultant degranulation, with the release of a host of inflammatory mediators that are, in large part, responsible for allergic nasal symptoms that include sneezing, rhinorrhea, itching, and nasal congestion. Other proinflammatory sub-stances are also generated after antigen exposure, most prominent are eosinophil products and cytokines. Cytokines are thought to be generated in part by lymphocytes, which are abundant in resting and stimulated nasal mucosa, and also by mast cells that have an important role in the storage and production and secretion of cytokines. Cytokines will up-regulate adhesion molecules on the vascular endothelium, and possibly on marginating leukocytes, and lead to the migration of these inflammatory cells into the site of tissue inflammation. Various cytokines will also promote the chemotaxis and survival of these recruited inflammatory cells and lead to a secondary immune response by virtue of their capability to promote IgE synthesis by B cells. Also important is the nervous system, which amplifies the allergic reaction by central and peripheral reflexes that result in changes at sites distant from those of antigen deposition such as the eye, sinuses and lower airway. These inflammatory changes lower the threshold of mucosal responsiveness to various specific and nonspecific stimuli, making allergic patients more responsive to stimuli to which they are exposed every day.

The pathophysiologic mechanisms thought to be involved in the generation of ocular symptoms in patients with AR deserve special attention. It is likely that these symptoms result both from the direct effects of allergen deposition on the conjunctiva but also because of nasal ocular reflexes. In support of direct allergen deposition resulting in the symptoms is the fact that ocular allergen challenge leads to symptoms of watery and itchy eyes that are associated with the release of inflammatory mediators, including histamine, in ocular secretions[18,19]. In support of nasal ocular reflexes is the fact that nasonasal reflexes, that is, the generation of symptoms in one nasal cavity in response to stimulation of the other cavity, are well described and have been shown to occur in response to several stimuli including allergen, histamine, cold dry air, and capsaicin[20-24]. There is also evidence suggesting that nasal reflexes in response to allergen challenge lead to an inflammatory response in the maxillary sinus[25]. Therefore, it seems plausible that allergen depositing on the nasal mucosa can trigger afferent reflexes which then propagate centrally. The efferent limbs of these reflexes could then be propagated not only to the contralateral nasal cavity but also to both conjunctivae and maxillary sinuses. Another possible mechanism is that the nasal allergic reaction leads to the release of mediators from the nose and up-regulation of circulating cells, which, when attracted to the eye, are primed to release more mediators and cause more severe symptoms. One last possible mechanism of eye symptoms in AR is direct propagation of allergen from the nose to the eye via the nasolacrimal duct. This is not a likely mechanism as the direction of flow of secretions within the nasolacrimal duct is usually from the eye to the nose and not in the opposite direction. Furthermore, the orifice of the nasolacrimal duct in the nasal cavity is in the inferior meatus, well shielded by the inferior turbinate from external penetration by allergen.

## Intranasal Steroids

Many therapies are available for the treatment of AR. These include antihistamines (systemic and topical), decongestants (systemic and topical), leukotriene modifiers, anticholinergics, intranasal steroids, and combination therapies. Multiple studies comparing the different agents available for treatment indeed support the superior efficacy of intranasal steroids for allergic rhinitis[26,27]. As a consequence, the comprehensive role of intranasal steroids is well recognized as evidenced in both European and American guidelines for the treatment of allergic rhinitis[28,29].

Traditionally, allergic rhinitis clinical trials have focused on nasal symptoms; however, recent studies have highlighted the prevalence and significance of ocular symptoms and thus, the effect of intranasal steroids on ocular symptoms has been the focus of recent investigation. Indeed, studies have shown that intranasal steroids are effective in the control of ocular symptoms almost to the same degree that they positively impact nasal symptoms. In an early placebo-controlled, double blind, trial in subjects with seasonal allergic rhinitis, Settipane and colleagues showed that both total ocular and total nasal symptoms were significantly lowered after treatment with triamcinolone acetonide[30]. More recently, ocular symptoms have been included in many trials and more data has emerged to support the efficacy of intranasal steroids in the control of eye symptoms in allergic rhinitis.

In a retrospective analysis of pooled data from 7 randomized, double-blind trials that compared the efficacy of fluticasone propionate and placebo in seasonal allergic rhinitis in 1645 patients, intranasal fluticasone propionate was found to be significantly more effective than placebo in reducing the total ocular symptom scores after 1 and 2 weeks of treatment[31]. A similar post hoc analysis of the effect of mometasone furoate on ocular symptoms was performed on the results of a placebo controlled clinical trial performed in 353 subjects with seasonal allergic rhinitis[32]. The subjects recorded symptoms of ocular itching, tearing, and redness and these were combined into a total ocular symptom score (TOSS) for analysis. Mometasone furoate treatment resulted in a significant reduction from baseline in TOSS compared with placebo and the greatest improvement with individual symptoms occurred with tearing. Fluticasone furoate, a more recent addition to the intranasal steroid market in the United States, is also more effective than placebo in reducing ocular symptoms associated with seasonal allergic rhinitis. Comparison of once-daily intranasal fluticasone furoate with placebo in subjects with seasonal allergic rhinitis who had moderate-to-severe total ocular symptom scores at baseline concluded that mean reductions from baseline were significantly greater with fluticasone furoate than with placebo for total ocular symptom scores and each of the individual ocular symptoms[33]. Two other published studies support this conclusion[34,35].

Although the mechanism of action of intranasal steroid sprays in relieving ocular symptoms is not understood, several mechanisms have been proposed. By decreasing nasal inflammation, intranasal steroids may modulate or normalize the excess stimulation of reflex neural activity that occurs during allergic reactions, thereby reducing ocular symptoms. In addition, by inhibiting local nasal inflammation, that is, the production of cytokines and infiltration of inflammatory cells, intranasal steroids may have indirect systemic effects that reduce the recruitment of inflammatory cells in other tissues, including the eyes. This effect would be observed on the late response to ocular challenge with antigen. Some authors have suggested that intranasal steroids increase drainage in inflamed nasolacrimal ducts, thereby reducing conjunctival exposure to allergens and inflammatory mediators. However, duct patency has been found to be maintained in subjects who had symptomatic allergic responses after ocular challenge[36]. It has also been suggested that intranasal steroids might travel through the nasolacrimal duct, exerting their anti-inflammatory effect directly on the conjunctiva. However, the lack of steroid-related side effects such as glaucoma and cataracts[37,38] after prolonged use suggests that movement of intranasal steroids through the nasolacrimal duct is not a plausible mechanism for the ocular effects of these agents.

## The Nasal-Ocular Reflex Mechanism of Rhinoconjunctivitis and the Effect of Intranasal Steroids on the Reflex

To attempt to explain the beneficial effect of intranasal steroids on eye symptoms in AR, we focused on the nasalocular reflex response. Prior studies of the nasal ocular reflex after antigen stimulation have yielded mixed results. Lebel and colleagues, in a nasal-challenge study, reported that ~20% of allergic rhinitis sufferers experienced ocular symptoms during nasal provocation with grass pollen, suggesting that allergic ocular symptoms can occur without direct exposure of the conjunctiva to allergen[39]. Loth and Bende, on the other hand, concluded that nasal challenge with allergen does not increase lacrimal gland secretion, because inhibition of parasympathetic nerves by lidocaine did not reduce tears[40]. The conclusions of their study can be questioned because the placebo arm failed to demonstrate any significant increase in lacrimation after nasal challenge with allergen thus putting the value of the results obtained from the lidocaine arm of the study in doubt. Other studies using different forms of stimulation have supported the existence of a nasal-ocular reflex. Zilstorff-Pedersen reported bilateral lacrimation after unilateral irritation of the nasal mucosa[41]. Using capsaicin as a stimulant and as a desensitizer, Philip and colleagues showed that unilateral nasal challenge with capsaicin produced ocular tearing and watering. This was reduced significantly after repeated capsaicin challenges that led to desensitization of the response[24].

To determine whether nasal challenge with antigen induces a nasal-ocular reflex, we performed a double-blind crossover trial in 20 subjects with seasonal allergic rhinitis[42]. We speculated that histamine, released by mast cells upon allergen deposition on the nasal mucosa, initiated the afferent limb of the reflex response which resulted in contralateral nasal symptoms and also ocular symptoms within minutes of challenge. Therefore, we evaluated the effect of a topical antihistamine, azelastine, applied to the nasal cavity on the side of challenge on both the nasal and ocular reflex responses. Subjects were challenged with antigen in one nostril using filter paper discs, and the response was monitored in both nostrils and in both eyes. Symptoms were recorded. Discs (intranasally) and Schirmer strips (intraocularly) were used for collecting secretions in both nostrils and eyes and were weighed before and after collection allowing us to calculate the weight of generated nasal and ocular secretions, objective measures of rhinorrhea and watery eyes, respectively. The discs and Schirmer strips were then placed in buffer to allow elution of collected secretions and the supernatants were measured for levels of histamine, an indicator of mast cell activation, and albumin, a marker of vascular permeability.

**Figure 1:**
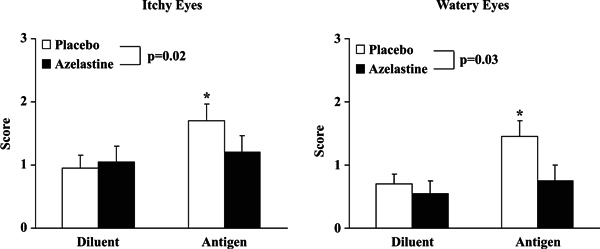
**Nasal-ocular reflex and inhibition by pretreatment with azelastine**. Watery eye and itchy eye symptom scores after diluent and allergen challenges. There was a significant increase in both itchy and watery eye symptoms after allergen challenge compared with respective diluent challenges with the patients on placebo depicted by the open bars (*P ≤ 0.004 vs respective diluents). Pretreatment with azelastine resulted in a significant reduction in the net change from the diluent response for both itchy (*P *= 0.02) and watery eye (*P *= 0.03) symptoms. (From Naclerio RM, Pinto J, deTineo M, Baroody FM. Elucidating the mechanism underlying the ocular symptoms associated with allergic rhinitis. *Allergy Asthma Proc*. 2008;29:24-28.)

Subjects were treated once topically at the site of challenge with azelastine or placebo. After placebo treatment, ipsilateral nasal challenge caused nasal symptoms and an increase in bilateral nasal secretion weights, both of which were blocked by treatment with azelastine. Levels of histamine and albumin increased only at the site of nasal challenge and azelastine inhibited the increase in albumin, but not that in histamine. These findings are not new and have been demonstrated by our and other laboratories previously. They cement the existence of a nasonasal reflex and the important role of histamine in its generation. As far as the ocular response, symptoms of itchy and watery eyes increased significantly after allergen challenge, compared with sham challenge, when the subjects were premedicated with placebo (Figure 1). This supports our hypothesis of the role of the naso-ocular reflex in the generation of ocular symptoms after allergen deposition on the nasal mucosa. Furthermore, the eye symptoms were inhibited by premedication with azelastine also suggesting that histamine, released by allergen challenge, was important in the genesis of the ocular symptoms (Figure 1). Ocular secretion weights increased bilaterally after placebo and were not inhibited by azelastine. Unfortunately, ocular secretion collection was technically difficult and ocular secretion weights are probably not as reliable an indicator of the ocular response as eye symptoms. This is related to the fact that the Schirmer strips led to irritation of the eyes and a high baseline of secretions even after the sham nasal challenge. In summary, the above data suggested that nasal allergen challenge induces histamine release at the site of the challenge, which causes both a nasonasal and a nasal ocular reflex. This antigen induced reflex is blocked by an H_1 _receptor antagonist applied at the site of the challenge. These observations support the hypothesis that eye symptoms associated with allergic rhinitis probably arise, at least in part, from a nasal-ocular reflex.

**Figure 2:**
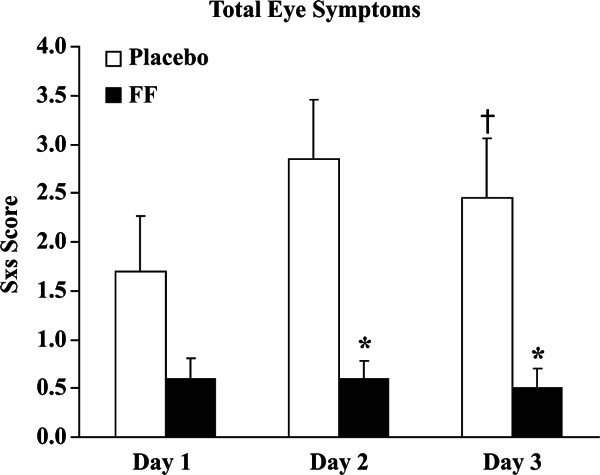
**Effect of fluticasone furoate nasal spray on total eye symptoms after allergen challenge**. The *x*-axis shows the days of consecutive challenge. Placebo responses are depicted in the open bars and fluticasone furoate (FF) in the solid bars. Net change from diluent is depicted as mean ± SEM (n = 20). †*P *≤ 0.04 versus day 1 on placebo treatment illustrating priming and *P < 0.01 versus placebo demonstrating the inhibitory effect of active treatment with FF.

To follow up on this study and investigate the effects of intranasal steroids on the nasal-ocular reflex, we performed double-blind, placebo-controlled, cross-over experiment in 20 subjects who had seasonal allergic rhinitis[43]. We hypothesized that repeated nasal allergen challenges would lead to priming and augmentation of nasonasal and nasal-ocular reflexes and that intranasal steroids would decrease inflammation and subsequently inhibit both nasonasal and nasalocular reflexes thus resulting in reduction of eye symptoms. Nasal antigen challenge was performed consecutively for 3 days after 1 week of treatment with either placebo or fluticasone furoate nasal spray. Subjects recorded their nasal and ocular symptoms, and nasal secretions were quantified. Nasal scrapings for quantifying eosinophils were obtained before each antigen challenge. When subjects were receiving placebo, nasal challenge with antigen led to sneezing, a nasonasal and a nasal-ocular reflex. Priming in the number of sneezes, contralateral nasal secretion weights, and total eye symptoms were observed (Figure 2). Pretreatment with fluticasone furoate nasal spray reduced sneezing, the nasonasal and nasal-ocular reflexes, and the amount of eosinophils in nasal secretions (Figure 2). The results of this study helped confirm the existence of a nasal-ocular reflex after allergen challenge of the nose, and demonstrated the exaggeration, or priming, of this reflex by repeated exposure to allergen and thus supported the role of the nasal-ocular reflex in the genesis of at least part of the eye symptoms in patients with AR. This study also helped demonstrate the efficacy of an intranasal steroid (fluticasone furoate) in reducing allergic inflammation, priming and subsequently the nasal-ocular reflex and ocular symptoms. Our results therefore support a mechanism that helps explain how control of eye symptoms can be achieved by the administration of an intranasal steroid in patients with seasonal allergic rhinitis.

## Conclusion

Eye symptoms (itching, watery eyes, and redness) are an important part of the overall burden of AR and are associated with significant bother to allergy sufferers. These effects probably occur by several mechanisms, the most obvious of which, is the direct deposition of allergen in the conjunctiva and the generation of an ocular inflammatory response. Another mechanism that might contribute to the genesis of ocular symptoms in allergic individuals is a neural reflex generated in the nose upon exposure to allergen that results in an amplification of the allergic response to the other nostril and also to both eyes. This mechanism might explain the efficacy of intranasally administered steroids in controlling ocular symptoms. In this article, we have reviewed evidence obtained both from experimental challenges and clinical studies that supports an important role for the nasalocular reflex in the eye symptoms of AR and, at least partially, explains the efficacy of intranasal steroids in controlling these symptoms.
